# Prescribable mHealth apps identified from an overview of systematic reviews

**DOI:** 10.1038/s41746-018-0021-9

**Published:** 2018-05-09

**Authors:** Oyungerel Byambasuren, Sharon Sanders, Elaine Beller, Paul Glasziou

**Affiliations:** Centre for Research in Evidence-Based Practice (CREBP), Bond University, Robina, QLD Australia

**Keywords:** Health care, Translational research

## Abstract

Mobile health apps aimed towards patients are an emerging field of mHealth. Their potential for improving self-management of chronic conditions is significant. Here, we propose a concept of “prescribable” mHealth apps, defined as apps that are currently available, proven effective, and preferably stand-alone, i.e., that do not require dedicated central servers and continuous monitoring by medical professionals. Our objectives were to conduct an overview of systematic reviews to identify such apps, assess the evidence of their effectiveness, and to determine the gaps and limitations in mHealth app research. We searched four databases from 2008 onwards and the Journal of Medical Internet Research for systematic reviews of randomized controlled trials (RCTs) of stand-alone health apps. We identified 6 systematic reviews including 23 RCTs evaluating 22 available apps that mostly addressed diabetes, mental health and obesity. Most trials were pilots with small sample size and of short duration. Risk of bias of the included reviews and trials was high. Eleven of the 23 trials showed a meaningful effect on health or surrogate outcomes attributable to apps. In conclusion, we identified only a small number of currently available stand-alone apps that have been evaluated in RCTs. The overall low quality of the evidence of effectiveness greatly limits the prescribability of health apps. mHealth apps need to be evaluated by more robust RCTs that report between-group differences before becoming prescribable. Systematic reviews should incorporate sensitivity analysis of trials with high risk of bias to better summarize the evidence, and should adhere to the relevant reporting guideline.

## Introduction

The number of smartphones worldwide is predicted to reach 5.8 billion by 2020^1^ and there are 6 million multimedia applications (apps) available for download in the app stores.^2^ According to the latest report from IQVIA Institute for Human Data Sciences (formerly IMS Institute for Healthcare Informatics) 318,000 of these are mHealth apps.^3^ As one of the prominent digital behaviour change interventions of our time, mHealth apps promise to improve health outcomes in a myriad of ways including helping patients actively measure, monitor, and manage their health conditions.^4^

Here, we propose a concept of “prescribable” mHealth apps, defined as health apps that are currently available, proven effective, and preferably stand-alone. When proven effective and available, stand-alone mHealth apps that do not require dedicated central servers and additional human resources, can join other simple low-cost non-pharmaceutical interventions that can be ‘prescribed’ by general practitioners (GPs).

However, although there are a number of systematic and other reviews of mHealth apps aimed at particular health conditions that examined different aspects of the apps such as the contents, quality and usability,^5–8^ no overview of systematic reviews has been done yet to summarize the effectiveness of stand-alone mHealth apps specifically, and across different health conditions that present in general practice. Overviews of reviews are an efficient way to gather the best available evidence in a single source to examine the evidence of effectiveness of interventions.^9^ Hence, our objectives were to: (1) conduct an overview of systematic reviews of randomized controlled trials (RCTs) to identify and evaluate the effectiveness of prescribable mHealth apps; and (2) determine the gaps and limitations in mHealth app research.

## Results

### Search results

**Fig. 1:**
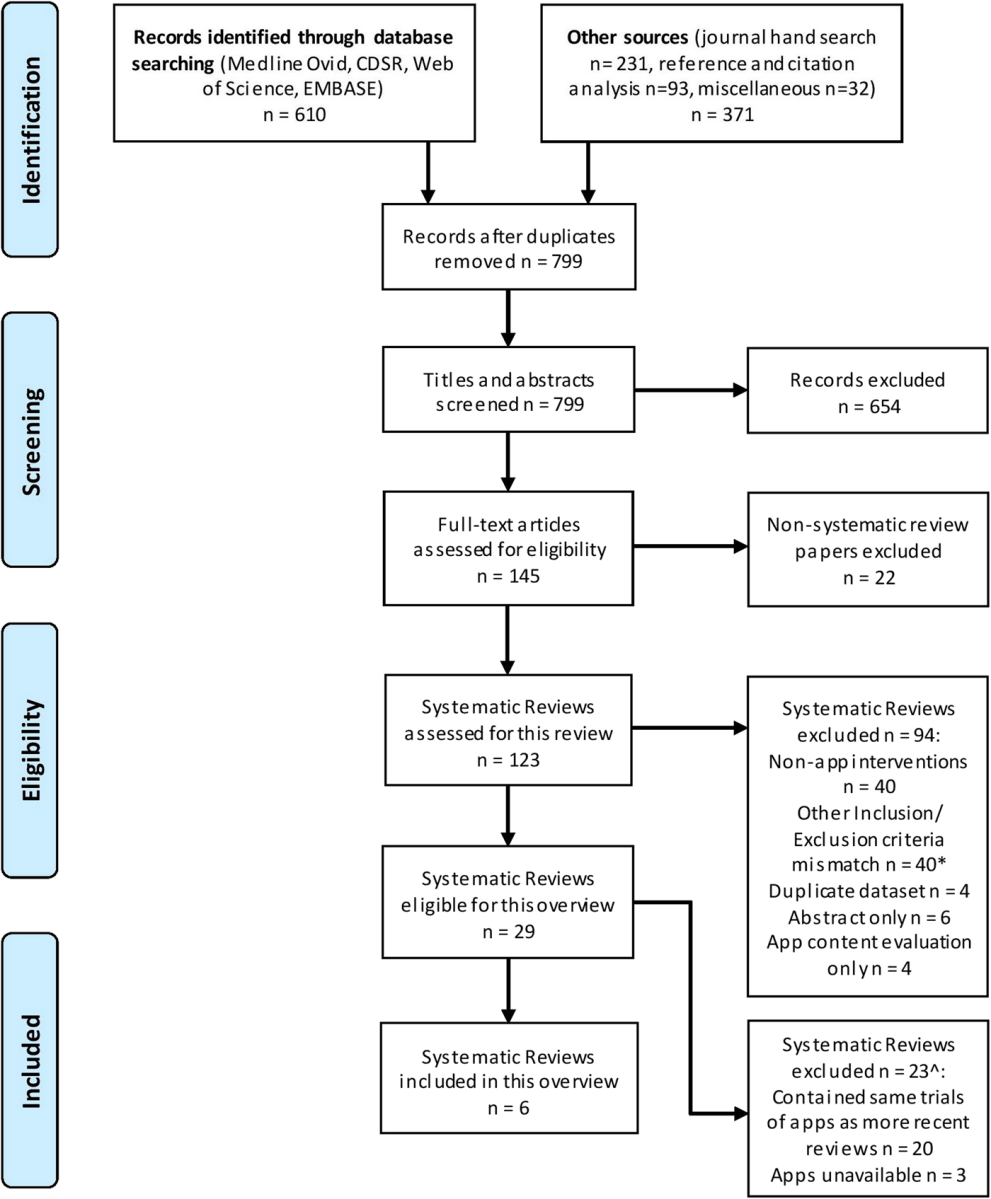
PRISMA flow diagram of selection of systematic reviews. *Table of excluded articles due to Inclusion and Exclusion criteria mismatch is provided as Supplementary Information 1. ^Table of excluded articles due to repeated coverage is provided as Supplementary Information 2

The PRISMA flowchart of the study selection process is presented as Fig. 1. Our electronic searches and the other sources search identified 981 publications. After deduplication, we screened 799 titles and abstracts, and assessed 145 full text articles for eligibility. One hundred and sixteen full text articles were excluded: 22 did not qualify as systematic reviews, 40 studies used non-app intervention, 4 studies were duplicates, 6 were abstracts only, 4 articles evaluated only the contents of the apps, and 40 studies did not meet one or more of the inclusion criteria (Supplementary Information 1). Of the 29 articles eligible for inclusion, 3 reviews were excluded due to apps still being unavailable and 20 reviews were excluded because they covered the same app trials as 6 more recent systematic reviews that were included in our overview (Supplementary Information 2).

**Fig. 2:**
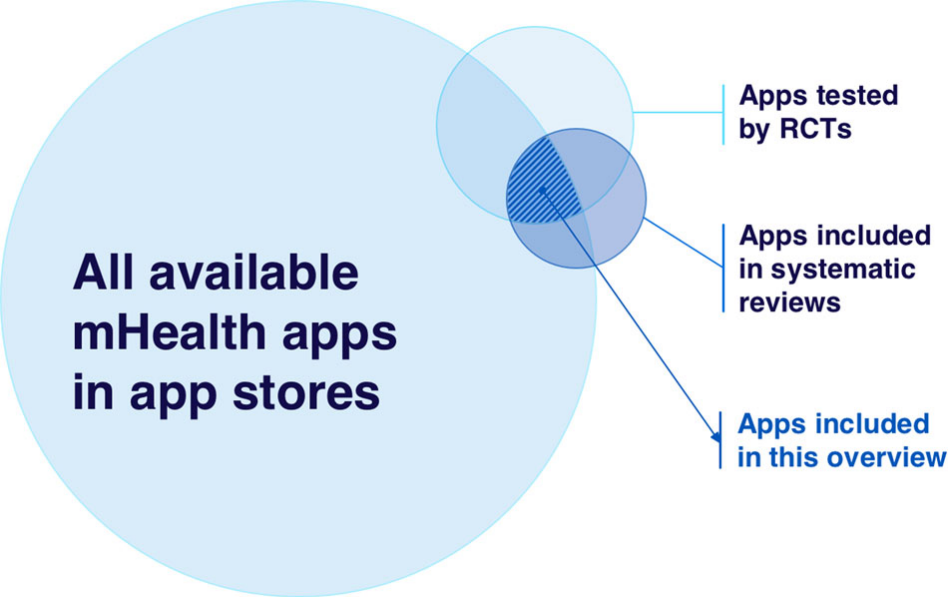
Scope of the overview

To achieve our study objectives, we used available systematic reviews of RCTs as a source of stand-alone mHealth apps that have been evaluated. We then determined the availability of those apps to ascertain the prescribability by searching the app stores and by contacting the authors of the RCTs. Figure 2 illustrates the scope of our study. Due to lack of established data on each category, the circle sizes and overlaps are illustrative.

We contacted 144 authors to determine the type and availability of their study apps. A little over half of the authors replied and we were able to include three app RCTs in our analysis as a result. We also found out that 25 app projects were discontinued.

### Description of included studies

Six published systematic reviews met the inclusion criteria for this overview.^10–15^ Characteristics and the limitations of the included systematic reviews are presented in Table 1. The systematic reviews were published between 2015–2017, and included a total of 93 RCTs and 18 studies of other designs. However, only 23 of the RCTs evaluated currently available stand-alone health apps. Characteristics of these RCTs are shown in Table 2 along with information about their availability and prescribability.

**Table 1:** Characteristics of the included systematic reviews

Review ID	Studies included	Participants/Population	Interventions	Comparison interventions	Reported outcome measures	Review limitations
Bonoto^10^ 2017, Brazil	13 RCTs^a^ (5 RCTs eligible for this overview)	Adults and children with DM	Mobile health apps	Any intervention	Blood glucose, HbA1c, total cholesterol, weight, HDL, LDL, triglycerides, BP, quality of life	Did not address the limitations of their review and how it compares with multitude of other similar diabetes app reviews. Minimal effort in evaluating and addressing the risk of bias in the primary studies. No sensitivity analysis was done to integrate risk of bias assessment to results.
Firth^11^ 2017, UK, USA, Australia	9 RCTs^a^ (2 RCTs eligible)	Adults with anxiety	Smartphone-supported psychological interventions to reduce anxiety	Waitlist, anti-anxiety medications, other non-smartphone interventions	Changes in aspects of anxiety	Risk of bias in the primary studies were addressed minimally. No sensitivity analysis was done to integrate risk of bias assessment into results.
Flores-Mateo^12^ 2015, Spain	9 RCTs^a^, 2 case-control studies (5 RCTs eligible)	Obese and overweight adults and children	Mobile apps that promote weight loss and increase in physical activity	Traditional intervention, paper hand-out, paper diary etc.	Body weight, BMI, and physical activity meta-analysis	The physical activity meta-analysis showed high heterogeneity (*I*^2^ = 93%), which was not explained by any sensitivity analysis. Minimal effort in evaluating and addressing the risk of bias in the primary studies. No sensitivity analysis was done to integrate risk of bias assessment to results.
Payne^13^ 2015, USA	14 RCTs^a^, 9 field, pilot or feasibility studies (7 RCTs eligible)	Adults	Mobile apps that disseminate health behaviour interventions	Unclear	Physical activity, diet, weight, alcohol consumption (binge drinking, frequency, blood alcohol concentration, etc.), HbA1c, sleep, stress, smoking, BMI	Lacked clearly defined comparator interventions and outcome measures. No risk of bias assessment was done for the included studies. Limitations of the review are addressed minimally.
Schoeppe^14^ 2016, Australia	20 RCTS^a^, 3 CTs, 4 pre-post studies (8 RCTs eligible)	Adults and children	Apps for influencing dietary intake, physical activity, sedentary behaviour	Unclear	Physical activity (MET min/week, steps, types, etc.), weight, BMI, BP, cardiorespiratory fitness, diet (fruit & vegetable servings), sedentary behaviour, etc.	Lacked clearly defined comparator interventions and outcome measures. Inappropriate use of CONSORT checklist to assess the quality of the primary studies. No risk of bias assessment was done for the included studies.
Simblett^15^ 2017, UK	39 RCTs^a^ (1 RCT eligible)	Adults with post-traumatic stress disorder (PTSD)	e-therapies	Waitlist or other active controls other than the intervention	PTSD (validated self-report or clinician-rated measures)	The primary meta-analysis showed high heterogeneity (*I*^2^ = 81%), which was not explained by the subgroup and sensitivity analysis. Only one of the included studies was a smartphone app therapy.

^a^Not all trials included in the systematic reviews were relevant for our overview purposes. Details of the eligible trials are provided in Table 2

**Table 2:** Characteristics of RCTs of prescribable apps

Study ID (Pilot?)	Population and length of follow up	Intervention	Comparator	Outcome	Availability and Prescribability
Bonoto 2017 systematic review. Five relevant RCTs out of 13.					
Berndt^16^ 2014, Pilot	Youth with T1DM (8–18 year olds, *n* = 68, 4 weeks)	Treatment with support of telemedicine system *Mobil Diab (mDiab)*	Conventional therapy only	Between-group differences in results were not reported, though both groups saw reduction in HbA1c. Intervention group had higher 'diabetes self-efficacy' score. Overall usability and acceptance were rated as 'good' (41%).	• Available in Germany in three versions: free stand-alone version mDiab Lite, full version mDiab €4.99, mDiab Pro version offers multi user license and connection to central web platform. • Lack of effectiveness is the barrier to prescribe this app.
Charpentier^17^ 2011	Adults with T1DM (*n* = 180, HbA1c > 8%, 6 months)	Group 1: quarterly visits + paper logbook Group 2: quarterly visits + *DiaBeo* app Group 3: biweekly teleconsultation + *DiaBeo* app (bolus calculator with validated algorithms)		At 6 months G3 patients had 0.91%, and G2 had 0.67% reduction in HbA1c compared to G1 (control) (*p* < 0.001). Also, patients in G1 and G2 spent 5 h more than G3 for office consultations.	• Available in France and reimbursed by the government. • Should it become available outside France and ongoing 700 patient trial results pending, it could be a prescribable app.
Drion^18^ 2015	Adults with T1DM (*n* = 63, 3 months)	*DBEES* app	Standard paper diary	No between-group differences were found in quality of life, HbA1c, daily frequency of self-measurement of blood glucose.	• Available worldwide free of charge. • Lack of effectiveness is the barrier to prescribe this app.
Holmen^19^ 2014	Adults with T2DM (*n* = 151, mean age 55.9–58.6, HbA1c > 7.1%, 1 year)	Group 1: TAU + *Few Touch app* Group 2:TAU + *Few Touch app* + nurse counselling for the first 4 months Group 3: TAU with GP		HbA1c level decreased in all groups, but did not differ between groups after 1 year. Those aged ≥ 63 years used the app more than their younger counterparts (OR 2.7; 95% CI 1.02–7.12; *p* = .045).	• Available in Norway, Czech and USA under the name Diabetes Dagboka (Diary). • Lack of effectiveness is the barrier to prescribe this app.
Kirwan^20^ 2013^a^	Adults with T1DM (*n* = 72, HbA1c > 7.5%, 6 months, +9 months follow up)	TAU + *Glucose Buddy* app + weekly SMS support	Usual care (3 monthly visit to healthcare provider)	There was a significant between group difference in HbA1c reduction (*p* < 0.001). At 9 months, the mean HbA1c reduction for intervention group was 1.10% (SD 0.74). The control group HbA1c increased slightly (mean 0.07, SD 0.99).	• Available worldwide free of charge. • It is prescribable for improving glycaemic control.
Firth 2017 systematic review. Two relevant RCTs out of 9.					
Pham^21^ 2016, Pilot	Adults with moderate anxiety (*n* = 63, 4 weeks)	*Flowy* app	Waitlist with weekly psychoeducation emails	There were no between-group differences in reductions in anxiety, panic and hyperventilation.	• Available worldwide free of charge. • Lack of effectiveness is the barrier to prescribe this app.
Roepke^22^ 2015	Adults with depression (*n* = 283, 4 weeks)	Group 1: *SuperBetter* app using CBT and positive psychotherapy (CBT-PPT SB) Group 2: General SuperBetter app Group 3: Waitlist control group		Group 1 and 2 achieved greater reductions in CES-D questionnaire scores than control by posttest (Cohen's d = 0.67) and by follow-up (d = 1.05).	• Available worldwide free of charge. • It could be prescribable. This trial suffered from high attrition (80%). Larger trials with longer follow ups are needed.
Flores-Mateo 2015 systematic review. Five relevant RCTs out of 9.					
Allen^27^ 2013^a^, Pilot	Obese adults (BMI > 28 kg/m^2^, *n* = 68, 6 months)	Group 1: Intensive counselling Group 2: Intensive counselling + *Lose It!* app Group 3: less intensive counselling +* Lose It!* app Group 4: *Lose It!*app alone		At 6 months, there was no statistically significant weight loss between the groups (mean weight loss in G1 was −2.5 kg, in G2 −5.4 kg, in G3 −3.3 kg and in G4 −1.8 kg, respectively.)	• Available worldwide. Basic version is free. • Lack of effectiveness is the barrier to prescribe this app on its own. Could be helpful as a support to weight loss counselling.
Carter^28^ 2013^a^, Pilot	Overweight adults (BMI > 27 kg/m^2^, *n* = 128, 6 months)	*MyMealMate* app	Self-monitoring slimming website OR calorie counting book by Weight Loss Resources company.	At 6 months, there was statistically significant difference in mean weight loss between app group (−4.6 kg) and website group (−1.3 kg) (*p* = 0.004), but not between app and diary group (−2.9 kg) (*p* = 0.12).	• Available worldwide free of charge. • It is prescribable for weight loss.
Glynn^38^ 2014^b^	Rural primary care patients (mean BMI 28.2 kg/m^2^, *n* = 90, 8 weeks)	*Accupedo-Pro* app and physical activity goal of 10,000 steps/day	Information on benefits of exercise and physical activity goal of walking for 30 min per day	There was a difference in mean improvement of 2017 (95% CI 265 – 3768, *p* = 0.009) steps per day between the groups, favouring the intervention. No significant changes were observed for secondary outcomes of blood pressure, weight and BMI.	• Available worldwide free of charge. • It is prescribable. An increase of over 1000 steps per day is clinically meaningful and, if continued, expected to result in long-term health benefits. Longer trials are needed to measure such effects.
Laing^29^ 2014	Adult primary care patients (BMI > 25 kg/m^2^, *n* = 212, 6 months)	*MyFitnessPal* app	Usual care + 'any activities you would like to lose weight'	At 6 months, there was no significant between group difference in weight change (−0.67 lb, 95% CI −3.3–2.11 lb, *p* = 0.63) and in self-reported behaviours around physical activity, diet and self-efficacy in weight loss.	• Available worldwide free of charge. • Lack of effectiveness is the barrier to prescribe this app on its own.
Turner-McGrievy^30^ 2011^a^	Overweight adults (BMI 32.6 kg/m^2^, *n* = 96, 6 months)	Weight loss podcast + *Fat Secret* calorie counter app and Twitter support group	Podcast only (same as intervention, twice a week for 3 months, and 2 minipodcasts a week for months 3–6)	Overall the two groups lost exactly the same amount of weight (−2.7 kg) and there was no significant difference in percentage weight loss between the groups (3.5% in intervention vs. 3.8% in control).	• Available worldwide free of charge. • Podcast was designed by study team and proven effective in their 2009 RCT. However, the app addition did not make any difference in the results. Lack of evidence to prescribe this app.
Payne 2015 systematic review. Seven relevant RCTs out of 14.					
Gajecki^24^ 2014	University students with risky alcohol consumption (mean age 24.7, *n* = 1929, 7 weeks)	Group 1: smartphone app *Promillekoll* Group 2: web-based app *PartyPlanner* that calculates blood alcohol concentration (BAC)	No intervention	Per-protocol analyses revealed only one significant time-by-group interaction, where Group 1 participants increased the frequency of their drinking occasions compared to controls (mean at baseline 2.24/wk, mean at FU 2.36/wk, *p* = 0.001).	• Available in Sweden. • This study showed youth drinking behaviour needs to be explored further and apps need to provide more in-depth information than just the BAC. Such apps are not prescribable as they are.
Gustafson^25^ 2014	Alcohol dependent adults leaving residential programs (mean age 38.3, *n* = 349, Intervention 8 months + follow up 4 months)	TAU + Addiction-Comprehensive Health Enhancement Support System (*A-CHESS*) app	Treatment as usual (TAU) (no continuing care)	*A-CHESS* group reported fewer risky drinking days than the control group, with a mean of 1.39 vs 2.75 ds/previous 30 ds (mean difference, 1.37; 95%CI 0.46–2.27; *p* = 0.003).	• Available in the USA through the agency involved with the study. • Should this app be made widely available, it can be prescribed to help with continuing care for people leaving residential programs and generally for people with alcohol dependency.
Watts^23^ 2013, Pilot	Adults with mild and moderate depression (*n* = 35, 8 weeks, +3 month follow up)	*The Sadness Program* CBT lessons adapted for *Get Happy* app	Same content on a website (previously proven effective)	The results indicate that reductions in PHQ-9, the BDI-II and K-10 pre- to post-intervention and pre- to follow up, were significant, regardless of experimental group.	• Available in Australia as Managing depression for AUD 59.99. • It is prescribable. The price could be a barrier for widespread use. It needs to be tested in a larger trial.
Schoeppe 2016 systematic review. Eight relevant RCTs out of 20.					
Cowdery^32^ 2015	Adults (*n* = 40, 12 weeks)	Choice of either *Zombies! Run or The Walk* + *MOVES* app	MOVES (activity monitoring app)	There were no significant between-group differences in physical activity, enjoyment of exercise and motivation to exercise.	• All apps are available worldwide free of charge. • Lack of effectiveness is the barrier to prescribe these apps.
Dirieto^33^ 2015	Insufficiently active healthy young people (14–17 years old, *n* = 51, 8 weeks)	Group 1: Immersive exergame app *Zombies! Run* Group 2: non-immersive app *Get Running*	No intervention	There were no significant between-group differences in cardiorespiratory fitness (1-mile walk/run test) and self-reported physical activity levels and its predictors.	• Both apps are freely available. • Lack of effectiveness is the barrier to prescribe this app.
Mummah^34^ 2016, Pilot	Overweight adults (BMI 32 kg/m^2^, *n* = 17, 12 weeks)	*Vegethon* app (to monitor vegetable consumption, set goals, get feedback and comparison)	Waitlist	Intention to treat analysis at the end of 12 weeks showed the between group vegetable consumption difference was 7.4 servings a day (95% CI 1.4–13.5; *p* = 0.02).	• Available worldwide free of charge. • This study was done on select participants of a 12-month weight loss program. It is prescribable; however, larger trial is needed.
Silviera^35^ 2013, VanHet Reve^36^ 2014	Autonomous-living seniors (mean age 75, *n* = 44, 12 weeks)	Group 1: *ActiveLifestyle* tablet app for strength-balance exercises, social motivation version Group 2: *ActiveLifestyle* tablet app, individual motivation version Group 3: brochure-based intervention		Between group comparison showed moderate improvement for gait velocity and cadence in tablet groups. Social motivation strategies proved more effective than individual strategies in stimulating the participants.	• Available in Italy free of charge. • The study measured surrogate outcomes for falls prevention. Should it become available worldwide, it is prescribable to seniors and other people who need to improve their balance and gait.
Walsh^39^ 2016, Pilot	Young adults (17–26 years old, *n* = 55, 5 weeks)	Aim for 10,000 steps/d using *Accupedo-Pro* pedometer app with feedback and goal setting functions	Information on daily recommended physical activity and told to walk for 30 min/day	Between group differences revealed intervention group increased daily steps significantly (2393, about a mile) more than those in the control group (1101; *t*_53_ = 2.07,* p* = 0.043).	• Available worldwide free of charge. • This app is prescribable to those who are interested in improving their physical activity levels by walking.
Wharton^37^ 2014, Pilot	Adults with BMI 25–40 kg/m^2^, (*n* = 57, 8 weeks)	Group 1: record food intake using *Lose-It!* app Group 2: record food intake using phone’s memo function Group 3: record using paper-and-pencil method		There was no between group differences in weight loss, BMI, and Healthy Eating Index at the end of study. The app group lost a slightly less weight (−3.5 lb) than the other groups (G2 lost −6.5 lb and G3 lost −4.4 lb).	• Available worldwide. Basic version is free. • This app could be prescribed as food intake recording tool. Larger and longer trials are needed to establish advantage of using this app in weight loss interventions.
Simblett 2016 systematic review. One relevant RCT out of 39.					
Miner^26^ 2016, Pilot	Adults with PTSD symptoms (*n* = 49, 2 months)	*PTSD Coach* app	Waitlist (crossed over after 1 month)	There was no statistically significant between group differences in PTSD scores according to the PTSD Checklist—Civilian (PCL-C). The app was deemed acceptable and the intervention feasible.	• Available worldwide free of charge. • Lack of effectiveness is the barrier to prescribe this app. Larger and longer trials are needed.

*TAU* treatment as usual, *CES-D* Center for Epidemiological Studies Depression Questionnaire, *BMI* body mass index, *MET* metabolic equivalent of task, *PHQ-9* Patient Health Questionnaire 9, *BDI-II* Beck’s Depression Inventory Second edition, *K-10* The Kessler 10-item Psychological Distress scale, *PTSD* posttraumatic stress disorder

^a^These studies are also included in Payne 2015 systematic review

^b^ These studies are also included in Schoeppe 2016 systematic review

One of the systematic reviews addressed diabetes,^10^ two addressed mental health,^11,15^ another two addressed physical activity and weight loss related issues,^12,14^ and one addressed all of these areas by addressing the behavior change aspect of apps.^13^ Four of the reviews also included meta-analyses.^10–12,15^ We described the systematic reviews and the RCTs in further detail under thematic subheadings.

### Effects of interventions

#### Diabetes

The Bonoto 2017 systematic review assessed app interventions for diabetes mellitus type 1 and 2^10^. It included 13 RCTs, of which 5 were relevant to this overview.^16–20^ All of the RCTs included apps that aimed to improve glycemic control and quality of life as measured by multiple biochemical markers. The meta-analysis showed a mean difference of −0.4% (95% CI −0.6, −0.3) in glycated haemoglobin levels favoring the intervention. Four trials tested apps for type 1 diabetes patients, of which two demonstrated a significant between group reduction in HbA1c levels.^17,20^ One trial that tested an app for type 2 diabetes patients did not show any between group differences in HbA1c levels at one year.^19^ All the diabetes apps include functions to log blood glucose levels, insulin dose, diet and physical activity, and to set push notifications and reminders. Two of the apps also offer versions for doctors to enrol and monitor multiple patients.^16,18^ At this stage, only two of these diabetes apps are available free of charge worldwide,^18,20^ and the other three apps are available either in Germany, France, or Norway (Table 2).

#### Mental health

The Firth 2017 systematic review assessed interventions aimed at reducing anxiety.^11^ It included nine RCTs, of which two were relevant to this overview. Their meta-analysis of the effects of smartphone interventions on symptoms of anxiety found small-to-moderate positive effect favoring the intervention (Hedges’ *g* = 0.3, 95% CI 0.2, 0.5).

Two of the RCTs from this review used stand-alone apps that were available. A breathing retraining game app called *Flowy* did not show any significant reduction in anxiety, panic, and hyperventilation.^21^ The basic version of *SuperBetter* app was tested against its “fortified” version, which contains more cognitive behavioral therapy (CBT) and positive psychotherapy content, and a waitlist control group.^22^ Depression scores were equally reduced in both app groups compared to the control group, but the attrition rate was high (80%) in both app groups over 4 weeks.

The Payne 2015 systematic review assessed app interventions for their behaviour change potential.^13^ It included 14 RCTs and 9 feasibility and pilot studies, of which 7 RCTs were eligible for our overview. Only one of the RCTs tested an app for depression against a previously validated web-based CBT program.^23^ Both groups had equally significant improvements. This app is now called *Managing Depression* as a part of 4 app series called *This Way Up* and available for AUD 59.99.

Two other trials included in Payne 2015 systematic review explored use of mobile apps to curb alcohol use among university students^24^ and patients leaving residential treatment for alcohol use disorder.^25^ The results showed that alcohol use increased among university students who used the intervention app *Promillekoll*, which calculated blood alcohol concentration up to the legal limit.^24^ Whereas, the *A-CHESS* app that was designed to provide on-going support for people leaving alcohol rehabilitation was shown to reduce the risky drinking days in the previous 30 days (OR 1.94, 95% CI 1.14–3.31, *p* = 0.02).^25^ These apps are available in Sweden and the USA respectively.

The Simblett 2017 systematic review assessed e-therapies aimed at treating posttraumatic stress disorder (PTSD).^15^ It included 39 RCTs. The meta-analysis showed standardized mean difference of −0.4 (95% CI −0.5, −0.3) favoring the intervention in reducing the severity of PTSD symptoms, however the heterogeneity was high (*I*^2^ = 81), which was not explained by the subgroup and sensitivity analysis. Only one of the RCTs tested an app called *PTSD Coach* against waitlist control for 1 month; however, there were no significant between group differences in the PTSD Checklist–Civilian questionnaire result.^26^

#### Weight loss and physical activity

Two systematic reviews evaluated apps for weight loss and physical activity. The Flores-Mateo 2015 systematic review assessed studies aimed at increasing weight loss and physical activity for overweight and obese people of all ages.^12^ It included nine RCTs and two case control studies of which five RCTs were relevant to this overview. A meta-analysis of nine studies showed app interventions reduced weight by −1.0 kg (95% CI −1.8, −0.3) more than the control group. Net change in body mass index (BMI) showed mean difference of −0.4 kg/m^2^ (95% CI −0.7, −0.1) favoring the intervention. Net change in physical activity resulted in standard mean difference of 0.4 95% CI −0.1, 0.9), however, the heterogeneity was high (*I*^2^ = 93%) and the authors did not explain why several RCTs that reported physical activity outcomes were excluded from this meta-analysis.

Four of the RCTs from this review used calorie counting apps as interventions.^27–30^ However, only one of them (*MyMealMate* app) showed a statistically significant between-group difference in weight loss.^28^ The *MyMealMate* app includes calorie information of 23,000 UK-specific brands of food items in the database, and goal-setting, physical activity monitoring and automated text-messages functions. When compared against a self-monitoring slimming website, the app group lost notable amount of weight and BMI, but not compared to the control group that used a calorie counting paper diary. *MyFitnessPal* app is one of the consistently highest rated free apps for calorie monitoring and it contains database of 3 million food items. However, when tested on its own for 6 months, the intervention made almost no difference to the weight of the participants.^29^ This study also provided an insight on the usage of the apps during the trial, which showed that the logins to the app dropped sharply to nearly zero after 1 month from acquiring it. These three studies also suffered from a high overall attrition rate of more than 30% and the intervention groups lost more participants than the control groups. Another calorie-counting app *FatSecret* was tested as an addition to a weight-loss podcast made and previously proven effective by the same study team. The results showed no difference in weight loss between the groups.^30^

The Schoeppe 2016 systematic review assessed studies aimed at improving diet, physical activity and sedentary behavior.^14^ It included 20 RCTs, 3 controlled trials and 4 pre-post studies, but only 8 RCTs were relevant to this overview. It synthesized the trials in tabular and narrative formats, and assessed the quality of the trials using the CONSORT checklist.^31^ Two of the RCTs tested so-called 'exergame' (gamified exercise) apps called *Zombies! Run*, *The Walk*, non-immersive app *Get Running* and an activity monitoring app *MOVES*.^32,33^ Both studies had very low attrition rates, but failed to demonstrate any significant between group differences in improvements in physical activity and its indicators and predictors such as cardiorespiratory fitness, enjoyment of exercise and motivation.

One trial assessed an app aimed at increasing vegetable consumption called *Vegethon* on a small sample of participants of a 12-month weight loss program.^34^ People who used the app consumed more servings of vegetable per day than the control group at 12 weeks (adjusted mean difference 7.4, 95% CI 1.4–13.5; *p* = 0.02). Another physical activity trial tested a tablet-based app *ActiveLifestyle* among independently living seniors.^35,36^ Between-groups comparisons revealed moderate effect for gait velocity (Mann–Whitney *U* = 138.5; *p* = .03, effect size *r* = .33) and cadence (Mann–Whitney *U* = 138.5, *p* = .03, effect size *r* = .34) during dual task walking at preferred speed in favour of the tablet-based app groups.

There were two apps that were tested in two different studies included in both the Flores-Mateo and Schoeppe systematic reviews. The *Lose-It!* app was tested for 6 months^27^ and for 8 weeks.^37^ Not only was there no difference in weight loss between groups, in the second study the app group lost less weight than the two control groups that used a paper diary and the memo function of the phone. In contrast, the *AccupedoPro* pedometer app demonstrated a similar amount of increase in daily steps both in general primary care patients^38^ and in young adults.^39^

### Risk of bias in included systematic reviews

The overall results of risk of bias in the six systematic reviews evaluated by the Cochrane risk of bias in systematic reviews (ROBIS) tool^40^ is presented in Table 3. Overall, five of the reviews had high risk of bias and one had low risk. ROBIS assessment has three phases: the first one (optional) assesses the relevance of included reviews to the overall review question (not reported here). The second phase evaluates detailed risk of bias in four domains (Table 3). The first domain (study eligibility criteria) revealed that none of the systematic reviews had a published protocol specifying their eligibility criteria and analysis methods. However, the detailed information provided in their methods sections regarding eligibility and analyses, combined with the rest of the domain questions made it possible for us to evaluate the whole domain low risk of bias for all the reviews. The main issue with the second domain (study identification and selection) was limiting the literature search to only English language publications. We considered this to be a serious hurdle in retrieving as many eligible studies as possible because many Spanish and Portuguese speaking countries as well as many European countries are conducting and publishing mHealth research actively. The third domain (data collection and study appraisal) had issues around lack of information about the effort to minimize error in data collection and failure to formally assess the risk of bias in the primary studies. In the last domain of phase 2 (synthesis and findings), the reviews received “no” on reporting of all pre-defined analysis or explaining departures due to lack of published protocols, relating back to domain 1. Also, the risk of bias levels in the primary studies was neither minimal nor sufficiently addressed in the synthesis in all but one review.

**Table 3:**
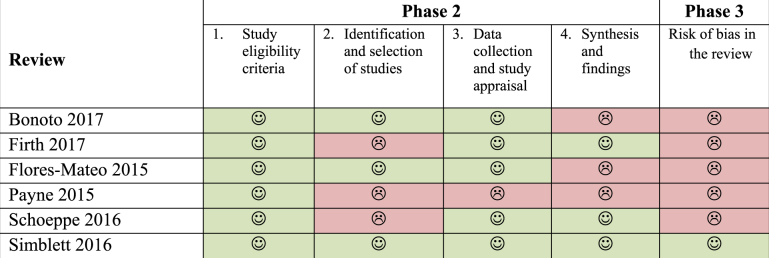
Overall results of risk of bias in systematic reviews (ROBIS) assessment

Phase 3 assesses the overall risk of bias of the systematic review. The main issue in this summary of risk of bias was with the first signalling question asking if the reviews addressed the concerns identified in the previous four domains in their discussions. All studies failed to recognize and address the potential sources of risk of bias that were identified in the domains of phase 2.

## Discussion

### Principal finding

Our overview evaluated six systematic reviews that included 23 RCTs of 22 currently available stand-alone health apps. Eleven of the 23 trials showed a meaningful effect on health or surrogate outcomes attributable to apps (Table 2). However, the overall evidence of effectiveness was of very low quality,^41^ which hinders the prescribability of those apps. Most of the app trials were pilot studies, which tested the feasibility of the interventions on small populations for short durations. Only one of the pilot trials has progressed on to a large clinical trial.^17^ The most commonly trialed apps have been designed to address conditions with the biggest global health burden: diabetes, mental health, and obesity. Although there is widespread acceptance of smartphones and promise of health apps, the evidence presented here indicates few effectiveness trials of health apps have been conducted. The risk of bias of both the included reviews and the primary studies is high. The reviews lacked sensitivity analyses to integrate the risk of bias results into context. Some of the RCTs also suffered from high attrition rates, and sometimes attrition was greater in the intervention group than the control group^21,22^; thus compromising the positive results and the conclusions drawn from the studies.

### Strengths

Although we set out to do a traditional overview of systematic reviews, it quickly became apparent that in order to ascertain the availability of the stand-alone mHealth apps, which was crucial to our objectives, we needed to investigate the primary trials evaluating the apps. We have provided a window into the body of evidence on currently available stand-alone mHealth apps with a special focus on the 'prescribability' in general practice settings, because this is where effective stand-alone apps can benefit both general practitioners and patients. It is also possible for other primary care practitioners, such as diabetes nurses and physiotherapists, to prescribe suitable health apps to patients.

There are a number of previous overviews of systematic reviews in eHealth and mHealth areas that can be comparable to ours in scope and methodology.^42–46^ Two of these used the Overview Quality Assessment Questionnaire and the others used the AMSTAR tool to assess the quality of the included systematic reviews.^47,48^ We chose to use Cochrane’s newly developed ROBIS tool, which focuses more on the risk of bias attributes and the quality of the methods compared to AMSTAR.^49^ Also, we did not restrict our search to any one language as many of the overviews did. Overviews are often limited by the individual limitations of the included systematic reviews, lack of risk of bias assessments, and challenge of synthesizing the overall results, and ours is no exception. We sought to overcome these limitations by contacting an extensive list of primary study authors to fill in the gaps left by the systematic reviews, and by assessing the risk of bias of included reviews vigorously. Despite these differences in methodology, our findings echo the conclusions of all the overviews regarding low quality of evidence in mHealth and eHealth areas they investigated. However, each of these overviews covered mixture of interventions, ranging from text messages, web tools, phone calls to apps, making them general and broad. We aimed to make our study more useful by exclusively focusing on a specific type of mHealth intervention with a vision of practical application in general practice.

### Limitations

Our review was limited by the weaknesses in the systematic reviews we identified. The systematic reviews did not thoroughly adhere to the PRISMA statement^50^ by not assessing the included studies’ risk of bias or not integrating the risk of bias results into the overall synthesis, thus preventing the reader from recognizing the poor quality of the included studies. The lack of understanding of risk of bias assessment prevented the authors from addressing this limitation in their discussions, as was evident during our ROBIS assessment. In addition, our overview was unable to assess the RCTs of health apps published in the past year because they are yet to be included in any systematic reviews and we specifically aimed to synthesize only systematic reviews. This highlights the necessity of timely updates of high levels of evidence in this field, which is further discussed in the ‘Implications for research’ section.

Furthermore, information regarding app availability was often not available in the primary studies. Thus, to compile the information on practical issues in Table 2 and to determine the current availability of apps, we had to contact primary study authors and search in the app stores. This emphasizes the importance of providing complete and transparent reporting of app interventions,^51^ as is true of other interventions in health care.^52^ We believe that sharing information amongst researchers working in app development is vital to reduce research waste and prevent re-invention of wheels.^53^ We also found several cases where, despite the initial trials failing to demonstrate any positive benefit, the apps were still released (Table 2), adding to the ‘noise’ rather than the ‘signal’ in this field, and leading to opportunity costs. In other cases, app testing and release were terminated due to lack of ongoing funding as the technology requires constant updates and improvements. Thus, it is important to secure a necessary funding source before engaging in an app development and testing efforts.

### Implications for practice

At present, anyone can create and publish health and medical apps in the app stores without having to test them, and patients must experiment with apps by trial and error. If GPs are to prescribe health apps, then they must be confident that the apps are shown to work, have fair privacy and data safety policies, and are usable at the very least. However, both assessment of individual apps and literature searches on app evidence are highly time-consuming and challenging for doctors to do on their own. Hence, we suggest that an independent and reliable source to carry out the evaluation of apps and to provide a collection of trustworthy mHealth apps is vital in providing doctors with prescribable apps.

The recently re-opened NHS Apps library is a great example of such source of apps for doctors’ use, despite the initial hurdles with the data safety of some of their previously recommended apps.^54^ They now employ a US-based app called *AppScript*, which contains all the apps in the NHS App library, to make app prescribing even easier for doctors.^55^ There have been numerous efforts around the world to provide quality and efficacy assessments of mHealth apps, each devising and using their own app evaluation framework. The challenges and the complexity of those efforts are well summarized by Torous et al.^56^ Thus, we believe initiatives like NHS App library are the safer and more accountable way to implement digital interventions in real practice. Like clinical guidelines, a recognized national body can decide what framework they want to use to evaluate apps and which apps to deem safe for use in practice in that particular country.

### Implications for research

Our overview found a number of methodological shortcomings in evaluation of mHealth apps. Consistent sources of high risk of bias in the primary RCTs were failure to blind participants and personnel to the intervention, as well as poor reporting of allocation concealment. Although blinding can be challenging in mHealth studies, it is important because of the digital placebo effect.^57^ Creating and using a basic static app or sham app for the control groups can help account for digital placebo effect and help establish the true efficacy of the interventions. Allocation concealment in mHealth trials can be done in the same way as in any other RCT by employing personnel who do not have any contact with the participants to handle the app installations; however, hardly any RCTs tried to ensure this. Several studies also noted that the control groups were susceptible to contamination with apps using the same or similar interventions to the tested app, since there are thousands of apps freely available to them outside of the research setting.^29,58^ A solution to this issue would be to increase the sample size to allow for drop-ins to the study intervention or similar ones, and to measure the usage of the apps to assess the contamination. Lastly, the only way to establish the effect of an intervention is by demonstrating greater change in one group compared to the other, rather than comparing it to the baseline.^59^ Yet, many RCTs failed to report their results as between-group differences and to adhere to the relevant guideline.^31^

The value of RCTs to evaluate fast-evolving mHealth interventions has been challenged due to their long duration, high cost and rigid designs. Although multitude of modifications and alternative methods have been suggested, widespread consensus is yet to be reached.^60–62^ As our overview showed, the effect of apps as health interventions might be marginal, and such small benefits can only be reliably detected by rigorous testing. Thus, RCTs should remain the gold standard, but should be employed strategically, and only used when the intervention is stable, can be implemented with high-fidelity, and has a high likelihood of clinically meaningful benefit.^63^

We also emphasize the value of traditional systematic and other reviews. The role of these higher levels of evidence is not only to assess and summarize the evidence in a field, but also to reveal the gaps and shortcomings in existing research, which our overview has done. If a review finds that the base of the evidence pyramid is shaky, that is the trials being done are not of high quality, then we must endeavour to fix it. The traditional reviews are also incorporating new technology. The Cochrane Collaboration’s recent advance in the area of living systematic reviews that are 'continually updated, incorporating relevant new evidence as it becomes available', offers significant opportunity to reduce the amount of time and effort it takes to update high level evidence.^64,65^ This will be invaluable in digital health research and evidence base building. As the supporting technologies of automation and machine learning continue to improve and become widespread, more time and human effort will be saved, and the easier it will be to update the evidence.^66^

## Conclusion

Smartphone popularity and mHealth apps provide a huge potential to improve health outcomes for millions of patients. However, we found only a small fraction of the available mHealth apps had been tested and the body of evidence was of very low quality. Our recommendations for improving the quality of evidence, and reducing research waste and potential harm in this nascent field include encouraging app effectiveness testing prior to release, designing less biased trials, and conducting better reviews with robust risk of bias assessments. Without adequate evidence to back it up, digital medicine and app 'prescribability' might stall in its infancy for some time to come.

## Methods

The Preferred Reporting Items for Systematic reviews and Meta-Analyses (PRISMA) reporting guideline and the Overview of Reviews chapter (Chapter 22) of the Cochrane Handbook of Systematic Reviews of Interventions Version 5.1.0 were used as general guides to conduct this overview.^50,67^

### Inclusion criteria

We included systematic reviews that evaluated at least one RCT of a currently available stand-alone health app. When the systematic review included other types of primary studies as well as RCTs, we reported only on the results of the RCTs. Our inclusion criteria are summarized in Table 4.

**Table 4:** Summary of inclusion criteria

Population	Patients of all ages, gender and races, with any type of health conditions
Intervention	Stand-alone smartphone or tablet apps that are readily available from leading app stores
Comparison	No intervention, treatment as usual, traditional or paper-based interventions, waitlist, or another recognized treatment
Outcome	Objective measurable health outcomes (e.g., reduction in HbA1c, waist circumference, BMI or weight loss), quality of life outcomes and mood and behaviour changes reported according to relevant and validated questionnaires (e.g., Depression and Anxiety Stress Scale (DASS)).
Study design	Systematic reviews of RCTs (if the systematic reviews included other study designs, we will only report on the results of the relevant RCTs)
Time limit	Systematic reviews published from 2008 and onwards

### Exclusion criteria

We excluded systematic reviews if they did not include any RCTs (i.e., included only case-control or cohort or other observational studies), included RCTs of apps that are not stand-alone or currently available; focused only on content evaluation of apps; reported no measurable health outcome; or were feasibility trials of app development; and used the following interventions: text or voice messages; apps aimed at health professionals; appointment and medication reminder apps; PDAs, video games, consoles, or other devices; or only native smartphone features such as built-in GPS and accelerometer. We also excluded study protocols and conference abstracts, of which the full text articles were not found.

### Search methods

#### Electronic database searches

We searched four electronic databases for systematic reviews without language restrictions: Medline Ovid, Cochrane Database of Systematic reviews, EMBASE, and Web of Science from 1 January 2008 through 1 February 2017. The cut-off date of 2008 was chosen as it coincides with the release of smartphones capable of running third-party Apps and when the two major App stores opened. We developed the initial search terms for Medline Ovid, and then modified them for other databases. Our search terms included combinations, truncations, and synonyms of 'cell phone', 'smartphone', 'application', 'intervention', 'patient', 'public', 'outcome', 'effectiveness', 'improvement', 'reduction', 'review' and 'meta-analysis'. The full search strategy for all databases is provided as Supplementary Information 3.

#### Searching other resources

In addition to the search of electronic databases, we did forward and backward citation searches of included systematic reviews, and hand-searched the *Journal of Medical Internet Research (JMIR)* from inception. We also contacted the authors of potentially includable trials to ascertain the availabilities and the progress of the app interventions as it was often unclear whether the apps were released, discontinued, or still in testing with plans for release. Additionally, we contacted many authors of trials that used text messages, PDA apps and web-based interventions to find out if those interventions were developed into smartphone apps.

### Data collection and analysis

#### Selection of reviews

Two authors (O.B., P.G.) screened titles and abstracts of the search results independently. We then retrieved in full text articles and one author (O.B.) assessed them according to the inclusion criteria outlined above with the second author (P.G.) assessing a random sample. Where the eligibility of the studies could not be determined due to insufficient information supplied in the abstract or absence of an abstract, the full text articles were obtained. Any disagreements between reviewers were resolved by discussion and consensus or by consulting with a third author (E.B.). When more than one publication of a study was found, the most recent and or the most complete one was used for data analysis. Systematic reviews excluded after full text review are provided as Supplementary Information 1 and 2 with reasons for exclusion.

#### Data extraction and assessment of risk of bias

Two authors (O.B., S.S.) independently extracted the following data from the included systematic reviews using a form developed by the authors for this review: study ID (first author’s last name and publication year), study characteristics (population, intervention, comparator, outcome, study design) and limitations of the review. We also extracted data from the RCTs of currently available stand-alone health apps. Along with general study characteristics information, we presented information gathered via contacting the authors for the availability of the intervention apps and other practical issues regarding their prescribability. Two authors (O.B., S.S.) assessed the risk of bias of the included systematic reviews according to Cochrane’s Risk of Bias in Systematic reviews (ROBIS) tool.^40^ Any disparities were resolved by consulting with a third author (E.B.).

### Data availability

All data generated or analysed during this study are included in this published article and its Supplementary Information files.

## Electronic supplementary material

Table of excluded articles due to inclusion and exclusion criteria mismatch(DOCX 23 kb)

Table of excluded articles due to repeated coverage(DOCX 19 kb)

Complete search strategy(DOCX 13 kb)

## Electronic supplementary material

**Supplementary information** accompanies the paper on the *npj Digital Medicine* website (10.1038/s41746-018-0021-9).
