# Supplementary material for: Prescribable mHealth apps identified from an overview of systematic reviews
Source: NPJ Digit Med. 2018 May 9;1:12. doi: 10.1038/s41746-018-0021-9 (PMC6550270; doi:10.1038/s41746-018-0021-9)
Supplement: Supplementary file 1 — Table of excluded articles due to inclusion and exclusion criteria mismatch(DOCX 23 kb) [file 41746_2018_21_MOESM1_ESM.docx]

**Appendix 2. Excluded systematic reviews due to Inclusion/Exclusion criteria mismatch**

| No. | Reference | Reason for exclusion |
| --- | --- | --- |
|  | Al-Durra, M., M.B. Torio, and J.A. Cafazzo, The Use of Behavior Change Theory in Internet-Based Asthma Self-Management Interventions: A Systematic Review. Journal of Medical Internet Research, 2014. 17(4). | This review did not report on the effectiveness of the interventions, but focused only on the theoretical base, guideline adherence and assessment tools. |
|  | Arambepola, C., et al., The Impact of Automated Brief Messages Promoting Lifestyle Changes Delivered Via Mobile Devices to People with Type 2 Diabetes: A Systematic Literature Review and Meta-Analysis of Controlled Trials. Journal of Medical Internet Research, 2016. 18(4). | This review did not include any stand-alone app studies. |
|  | Bort-Roig, J., et al., Measuring and influencing physical activity with smartphone technology: a systematic review. Sports Medicine, 2014. 44(5): p. 671-86. | None of the primary studies were RCT. They evaluated the feasibility, acceptability, and accuracy of smartphone accelerometers and pedometers. |
|  | Chomutare, T., et al., Features of Mobile Diabetes Applications: Review of the Literature and Analysis of Current Applications Compared Against Evidence-Based Guidelines. Journal of Medical Internet Research, 2011. 13(3). | The authors focused on the content of the apps, rather than study design and effectiveness of them. |
|  | Dale, L.P., et al., The effectiveness of mobile-health behaviour change interventions for cardiovascular disease self-management: A systematic review. European Journal of Preventive Cardiology, 2016. 23(8): p. 801-817. | Included one app prototype, but it was not stand-alone intervention. |
|  | de la Torre-Diez, I., et al., Cost-utility and cost-effectiveness studies of telemedicine, electronic, and mobile health systems in the literature: a systematic review. Telemed J E Health, 2015. 21(2): p. 81-5. | The authors’ aim wasn’t to evaluate measurable clinical outcomes. |
|  | de Souza, A.C.C., T.M.M. Moreira, and J.W.P. Borges, Educational technologies designed to promote cardiovascular health in adults: integrative review. Revista Da Escola De Enfermagem Da Usp, 2014. 48(5): p. 941-948. | The authors’ aim wasn’t to evaluate measurable clinical outcomes. Only one paper on development of physical activity app was included (not RCT). |
|  | DMBaron, J., H. McBain, and S. Newman, The impact of mobile monitoring technologies on glycosylated hemoglobin in diabetes: a systematic review. Journal of Diabetes Science & Technology, 2012. 6(5): p. 1185-96. | The authors specially interested in apps with real-time monitoring and feedbacks, which was our exclusion criterion. |
|  | Dute, D.J., W.J. Bemelmans, and J. Breda, Using Mobile Apps to Promote a Healthy Lifestyle Among Adolescents and Students: A Review of the Theoretical Basis and Lessons Learned. JMIR Mhealth Uhealth, 2016. 4(2): p. e39. | Measurable clinical outcome wasn’t the aim of this study. They focused on analysing the theoretical mechanisms used in apps. |
|  | El-Gayar, O., et al., A systematic review of IT for diabetes self-management: Are we there yet? International Journal of Medical Informatics, 2013. 82(8): p. 637-652. | The app studies included in this review were not stand-alone interventions. Also, measurable clinical outcome wasn’t the main aim of this review. |
|  | Fanning, J., S.P. Mullen, and E. McAuley, Increasing physical activity with mobile devices: a meta-analysis. J Med Internet Res, 2012. 14(6): p. e161. | This review did not include any RCT of available apps. Other app studies were not RCTs or the apps were discontinued. |
|  | Firth, J. and J. Torous, Smartphone Apps for Schizophrenia: A Systematic Review. JMIR MHealth and UHealth, 2015. 3(4): p. e102. | The outcomes reported in this study were of acceptability rating, and patient satisfaction levels, rather than clinically relevant measures. |
|  | Fitzner, K.K., et al., Telehealth Technologies: Changing the Way We Deliver Efficacious and Cost-Effective Diabetes Self-Management Education. Journal of Health Care for the Poor and Underserved, 2014. 25(4): p. 1853-1897. | This is more of an overview and did not include effectiveness studies. However, the included systematic reviews were also screened for eligibility. |
|  | Free, C., et al., The effectiveness of mobile-health technology-based health behaviour change or disease management interventions for health care consumers: a systematic review. PLoS Medicine / Public Library of Science, 2013. 10(1): p. e1001362. | The only app study included here was not a stand-alone intervention or available. |
|  | Fulford, H., et al., Exploring the Use of Information and Communication Technology by People With Mood Disorder: A Systematic Review and Metasynthesis. JMIR Ment Health, 2016. 3(3): p. e30. | They included only qualitative studies (focus group, interview etc). |
|  | Gandhi, S., et al., Effect of Mobile Health Interventions on the Secondary Prevention of Cardiovascular Disease: Systematic Review and Meta-analysis. Canadian Journal of Cardiology, 2016. 13: p. 13. | Included one app prototype, but it was not stand-alone intervention. |
|  | Greenwood, D.A., H.M. Young, and C.C. Quinn, Telehealth remote monitoring systematic review: Structured self-monitoring of blood glucose and impact on A1C. Journal of Diabetes Science and Technology, 2014. 8(2): p. 378-389. | Apps that were covered in this review were not stand-alone, and experimental. |
|  | Grist, R., J. Porter, and P. Stallard, Mental Health Mobile Apps for Preadolescents and Adolescents: A Systematic Review. J Med Internet Res, 2017. 19(5): p. e176. | This review included 2 RCTs of apps/prototypes, but the apps were discontinued. |
|  | Hidalgo-Mazzei, D., et al., Internet-based psychological interventions for bipolar disorder: Review of the present and insights into the future. Journal of Affective Disorders, 2015. 188: p. 1-13. | The outcomes reported in this study were of acceptability rating, retention and patient satisfaction levels, rather than clinically relevant measures. |
|  | Holtz, B. and C. Lauckner, Diabetes management via mobile phones: a systematic review. Telemedicine Journal & E-Health, 2012. 18(3): p. 175-84. | Apps that were covered in this review were not stand-alone and experimental. |
|  | Hui, C.Y., et al., The use of mobile applications to support self-management for people with asthma: a systematic review of controlled studies to identify features associated with clinical effectiveness and adherence. J Am Med Inform Assoc, 2016. | None of the apps included in this review were available. Most of the studies had web and SMS interventions. |
|  | Jackson, B.D., et al., EHealth Technologies in Inflammatory Bowel Disease: A Systematic Review. Journal of Crohn's & colitis, 2016. 10(9): p. 1103-21. | This review included one RCT protocol for an app study, and one non-stand-alone app study. |
|  | Joe, J. and G. Demiris, Older adults and mobile phones for health: A review. Journal of Biomedical Informatics, 2013. 46(5): p. 947-954. | This review included two experimental apps (since discontinued) and one non-stand-alone app study. |
|  | Karasouli, E. and A. Adams, Assessing the Evidence for e-Resources for Mental Health Self-Management: A Systematic Literature Review. JMIR Mental Health, 2014. 1(1): p. e3. | This review included one discontinued app study and one available app but study was not a RCT. |
|  | Levine, D.M., et al., Technology-assisted weight loss interventions in primary care: a systematic review. J Gen Intern Med, 2015. 30(1): p. 107-17. | Only one PDA-based app RCT was included. Eight other primary study authors were contacted for app development and availability, but none were made into an app. |
|  | Liang, X., et al., Effect of mobile phone intervention for diabetes on glycaemic control: a meta-analysis. Diabetic Medicine, 2011. 28(4): p. 455-63. | This review included one non- stand-alone app intervention. |
|  | Majeed-Ariss, R., et al., Apps and Adolescents: A Systematic Review of Adolescents' Use of Mobile Phone and Tablet Apps That Support Personal Management of Their Chronic or Long-Term Physical Conditions. Journal of Medical Internet Research, 2015. 17(12): p. e287. | The only app study included here was not a stand-alone intervention or available. |
|  | Marcano Belisario, J.S., et al., Smartphone and tablet self-management apps for asthma. Cochrane Database of Systematic Reviews, 2013. 11: p. CD010013. | Reviewed 2 RCTs that used asthma symptom diary type apps which weren’t stand-alone and discontinued. |
|  | Meurk, C., et al., Establishing and Governing e-Mental Health Care in Australia: A Systematic Review of Challenges and A Call For Policy-Focussed Research. J Med Internet Res, 2016. 18(1): p. e10. | This study did not aim to evaluate effectiveness of eHealth interventions, but to assess the research in regards to informing policy. |
|  | Mobasheri, M.H., et al., The uses of smartphones and tablet devices in surgery: A systematic review of the literature. Surgery, 2015. 158(5): p. 1352-71. | This review included one study, which used entertaining apps to distract kids waiting for surgery to reduce their anxiety. |
|  | Mosadeghi-Nik, M., M.S. Askari, and F. Fatehi, Mobile health (mHealth) for headache disorders: A review of the evidence base. Journal of Telemedicine & Telecare, 2016. 22(8): p. 472-477. | This review did not include any RCTs. All studies were of app developments. |
|  | Nelson, L.A., et al., Patterns of User Engagement with Mobile- and Web-Delivered Self-Care Interventions for Adults with T2DM: A Review of the Literature. Current Diabetes Reports, 2016. 16(7). | This review focused on patient engagement outcomes and associated technology features, but not clinical outcomes. |
|  | Pal, K., et al., Computer-based diabetes self-management interventions for adults with type 2 diabetes mellitus. Cochrane Database of Systematic Reviews, 2013. 0(3). | It covered two studies of non-stand-alone apps. |
|  | Park, L.G., et al., Mobile Phone Interventions for the Secondary Prevention of Cardiovascular Disease. Progress in Cardiovascular Diseases, 2016. 58(6): p. 639-50. | None of the app interventions are available. |
|  | Russell-Minda, E., et al., Health technologies for monitoring and managing diabetes: a systematic review. Journal of Diabetes Science & Technology, 2009. 3(6): p. 1460-71. | This review included one non-stand-alone app study. |
|  | Sarno, F., D.S. Canella, and D.H. Bandoni, Mobile health and excess weight: a systematic review. Revista Panamericana De Salud Publica-Pan American Journal of Public Health, 2014. 35(5): p. 424-431. | No real smartphone app studies were included (few PDA based or prototypes that were discontinued since). |
|  | Stephani, V., D. Opoku, and W. Quentin, A systematic review of randomized controlled trials of mHealth interventions against non-communicable diseases in developing countries. BMC Public Health, 2016. 16: p. 572. | This review included two studies that involved apps, but the apps were proprietary and discontinued afterwards. |
|  | Stephens, J. and J. Allen, Mobile phone interventions to increase physical activity and reduce weight: a systematic review. J Cardiovasc Nurs, 2013. 28(4): p. 320-9. | We contacted the authors of only one app RCT included in this review. However, it was discontinued after the study. |
|  | Versluis, A., et al., Changing Mental Health and Positive Psychological Well-Being Using Ecological Momentary Interventions: A Systematic Review and Meta-analysis. J Med Internet Res, 2016. 18(6): p. e152. | This review was aimed at evaluating the ecological momentary interventions usage in mHealth. It wasn’t app-specific and no clinical outcomes were reported. |
|  | Wesley, K.M. and P.J. Fizur, A review of mobile applications to help adolescent and young adult cancer patients. Adolescent Health Medicine & Therapeutics, 2015. 6: p. 141-8. | The studies included were not RCTs. They analysed contents of 6 applications and included feasibility studies. |
