# Supplementary material for: Prescribable mHealth apps identified from an overview of systematic reviews
Source: NPJ Digit Med. 2018 May 9;1:12. doi: 10.1038/s41746-018-0021-9 (PMC6550270; doi:10.1038/s41746-018-0021-9)
Supplement: Supplementary file 2 — Table of excluded articles due to repeated coverage(DOCX 19 kb) [file 41746_2018_21_MOESM2_ESM.docx]

**Appendix 3. Excluded systematic reviews due to repeated coverage**

| No. | Reference | Reason for exclusion |
| --- | --- | --- |
|  | Aguilar-Martinez, A., et al., Use of mobile phones as a tool for weight loss: a systematic review. Journal of Telemedicine and Telecare, 2014. 20(6): p. 339-349. | Includes one relevant RCT (Turner-McGrievy 2011), which is included in more recent systematic review (Flores-Mateo 2015) that is included on our overview. |
|  | Coughlin, S.S., et al., A Review of Smartphone Applications for Promoting Physical Activity. Jacobs J Community Med, 2016. 2(1). | Same search date and same includable studies as Flores-Mateo 2015. FM trumps by publication date. |
|  | Cui, M., et al., T2DM Self-Management via Smartphone Applications: A Systematic Review and Meta-Analysis. PLoS ONE [Electronic Resource], 2016. 11(11): p. e0166718. | Trumped by Bonoto 2017 (FTA) |
|  | Dallinga, J.M., et al., [Can apps encourage a healthier and more active lifestyle?]. Nederlands Tijdschrift voor Geneeskunde, 2016. 160(0): p. D329. | Trumped by Schoeppe 2017 (Cowdery 2015, Wharton 2014) |
|  | David, S.K. and M.R. Rafiullah, Innovative health informatics as an effective modern strategy in diabetes management: a critical review. International Journal of Clinical Practice, 2016. 70(6): p. 434-49. | Trumped by Bonoto 2017 (Diabeo, GB) |
|  | Deacon, A.J. and S. Edirippulige, Using mobile technology to motivate adolescents with type 1 diabetes mellitus: A systematic review of recent literature. Journal of Telemedicine & Telecare, 2015. 21(8): p. 431-8. | Includes only one app prototype RCT (Berndt 2014), which was also covered in more recent systematic review (Bonoto 2017) that is included on our overview. |
|  | Derbyshire, E. and D. Dancey, Smartphone Medical Applications for Women's Health: What Is the Evidence-Base and Feedback? International Journal of Telemedicine & Applications, 2013. 2013: p. 782074. | Included two relevant studies, which is included in more recent systematic review (Flores-Mateo 2015) that is included on our overview. |
|  | DiFilippo, K.N., et al., The use of mobile apps to improve nutrition outcomes: A systematic literature review. Journal of Telemedicine & Telecare, 2015. 21(5): p. 243-53. | As above |
|  | Donker, T., et al., Smartphones for smarter delivery of mental health programs: a systematic review. Journal of Medical Internet Research, 2013. 15(11): p. e247. | Includes one one app RCT (Watts 2013), which is included in more recent systematic review (Payne 2015) that is included on our overview. |
|  | Garabedian, L.F., D. Ross-Degnan, and J.F. Wharam, Mobile Phone and Smartphone Technologies for Diabetes Care and Self-Management. Current Diabetes Reports, 2015. 15(12). | The relevant RCTs were covered in more recent SR (Bonoto 2017), which we included in our overview. |
|  | Hamine, S., et al., Impact of mHealth chronic disease management on treatment adherence and patient outcomes: a systematic review. Journal of Medical Internet Research, 2015. 17(2): p. e52. | As above (Kirwan 2013 – Bonoto 2017) |
|  | Hood, M., et al., What do we know about mobile applications for diabetes self-management? A review of reviews. J Behav Med, 2016. 39(6): p. 981-994. | Trumped by Bonoto 2017 (FTA) |
|  | Hou, C., et al., Do Mobile Phone Applications Improve Glycemic Control (HbA1c) in the Self-management of Diabetes? A Systematic Review, Meta-analysis, and GRADE of 14 Randomized Trials. Diabetes Care, 2016. 39(11): p. 2089-2095. | Trumped by Bonoto 2017 (FTA, GB) |
|  | Khokhar, B., et al., Effectiveness of mobile electronic devices in weight loss among overweight and obese populations: a systematic review and meta-analysis. BMC Obesity, 2014. 1: p. 22. | (Carter 2013; Flores-Mateo 2015) |
|  | Lindhiem, O., et al., Mobile technology boosts the effectiveness of psychotherapy and behavioral interventions: a meta-analysis. Behavior Modification, 2015. 39(6): p. 785-804. | A-ChESS (Payne 2015), Glucose Buddy – (Bonoto 2017) |
|  | Liu, F., et al., The effect of mobile phone intervention on weight loss among overweight and obese adults: A meta-analysis of randomized controlled trials. Circulation, 2014. 130. | Includes one relevant RCT (Turner-McGrievy 2011), which is included in more recent systematic review (Flores-Mateo 2015) that is included on our overview. |
|  | McMillan, K.A., et al., A Systematic and Integrated Review of Mobile-Based Technology to Promote Active Lifestyles in People With Type 2 Diabetes. Journal of Diabetes Science & Technology, 2016. 21: p. 21. | Trumped by Bonoto 2017 (FTA) |
|  | O'Rourke, L., G. Humphris, and A. Baldacchino, Electronic communication based interventions for hazardous young drinkers: A systematic review. Neuroscience & Biobehavioral Reviews, 2016. 68: p. 880-90. | Only includes Promillekoll study which was covered in Payne 2015 |
|  | Whitehead, L. and P. Seaton, The Effectiveness of Self-Management Mobile Phone and Tablet Apps in Long-term Condition Management: A Systematic Review. J Med Internet Res, 2016. 18(5): p. e97. | Trumped by Bonoto 2017. |
|  | Wu, Y., et al., Mobile App-Based Interventions to Support Diabetes Self-Management: A Systematic Review of Randomized Controlled Trials to Identify Functions Associated with Glycemic Efficacy. JMIR Mhealth Uhealth, 2017. 5(3): p. e35. | Trumped by Bonoto 2017. |

**Excluded systematic reviews due to unavailability of apps**

| No. | Reference | Reason for exclusion |
| --- | --- | --- |
|  | Bassi, N., et al., Lifestyle Modification for Metabolic Syndrome: A Systematic Review. American Journal of Medicine, 2014. 127(12). | Most of the primary studies were of web, SMS and phone call interventions. Only one app prototype study (Spring 2012) was included, hence the app was not available yet. |
|  | Faurholt-Jepsen, M., et al., Electronic self-monitoring of mood using IT platforms in adult patients with bipolar disorder: A systematic review of the validity and evidence. Bmc Psychiatry, 2016. 16. | None of the apps are made available yet. RCTs still ongoing. |
|  | O'Reilly, G.A. and D. Spruijt-Metz, Current mHealth Technologies for Physical Activity Assessment and Promotion. American Journal of Preventive Medicine, 2013. 45(4): p. 501-507. | Only one app prototype RCT (Spring 2012) was included, hence the app was not available yet. |
