# Supplementary material for: Prescribable mHealth apps identified from an overview of systematic reviews
Source: NPJ Digit Med. 2018 May 9;1:12. doi: 10.1038/s41746-018-0021-9 (PMC6550270; doi:10.1038/s41746-018-0021-9)
Supplement: Supplementary file 3 — Complete search strategy(DOCX 13 kb) [file 41746_2018_21_MOESM3_ESM.docx]

**Final search strategy**

**Medline Ovid**

exp Cell phones/ OR exp Computers, Handheld/

AND

exp Mobile Applications/ OR (Application OR Applications OR App OR Apps OR Intervention OR Interventions).tw.

AND

exp Self care/ OR exp Life style/ OR exp Patient compliance/ OR exp Patient Care/

OR

(Smartphone OR Smart-phone OR Smart phone OR Smartphones OR Smart-phones OR Smart phones OR Mobile OR Tablet OR Tablets OR iPhone OR Android).tw.

ADJ2

(Application OR Applications OR App OR Apps OR Intervention OR Interventions).tw.

AND

(Utilize OR Utilizing OR Administer OR Administering OR Assist OR Manage OR Management OR Role OR Roles OR Tested OR Increase OR Increased OR Increasing).tw.

AND

exp Patients/ OR (Patient OR Patients OR Population OR Populations OR Public OR Group OR Groups OR Chronic OR Acute OR Behavior OR Behaviors OR Behavioral OR Behaviour OR Behaviours OR Behavioural).tw.

AND

exp "Outcome Assessment (Health Care)"/ OR (Outcome OR Outcomes OR Efficacy OR Effectiveness OR Improve OR Improved OR Improvement OR Improvements OR Reduce OR Reduced OR Reduction OR Reductions).tw.

AND

meta analysis.mp,pt.OR review.pt.OR search.tw. OR searched.tw. OR searches.tw.

**Cochrane Database of Systematic Reviews**

([mh "Cell phones"] OR [mh "Computers, Handheld"])

AND

([mh "Mobile Applications"] OR (Application OR Applications OR App OR Apps OR Intervention OR Interventions).tw.)

AND

([mh "Self care"] OR [mh "Life style"] OR [mh "Patient compliance"] OR [mh "Patient Care"])

OR

(Smartphone OR Smart-phone OR Smart phone OR Smartphones OR Smart-phones OR Smart phones OR Mobile OR Tablet OR Tablets OR iPhone OR Android):ti,ab

NEAR2

(Application OR Applications OR App OR Apps OR Intervention OR Interventions):ti,ab

AND

(Utilize OR Utilizing OR Administer OR Administering OR Assist OR Manage OR Management OR Role OR Roles OR Tested OR Increase OR Increased OR Increasing):ti,ab

AND

[mh Patients] OR (Patient OR Patients OR Population OR Populations OR Public OR Group OR Groups OR Chronic OR Acute OR Behavior OR Behaviors OR Behavioral OR Behaviour OR Behaviours OR Behavioural):ti,ab

AND

[mh "Outcome Assessment (Health Care)"] OR (Outcome OR Outcomes OR Efficacy OR Effectiveness

OR Improve OR Improved OR Improvement OR Improvements OR Reduce OR Reduced OR Reduction OR Reductions):ti,ab

**Embase**

('mobile phone'/exp OR ‘microcomputer'/exp)

AND

'Mobile Application'/exp OR (Application OR Applications OR App OR Apps OR Intervention OR Interventions):ti,ab

AND

('Self care'/exp OR 'Lifestyle'/exp OR ‘Patient compliance'/exp OR 'Patient Care'/exp)

OR

((smartphone OR 'smart phone' OR smartphones OR 'smart phones' OR mobile OR tablet OR tablets OR iphone)

NEAR/2

(application OR applications OR app OR apps OR intervention OR interventions)):ab,ti

AND

(Utilize OR Utilizing OR Administer OR Administering OR Assist OR Manage OR Management OR Role OR Roles OR Tested OR Increase OR Increased OR Increasing):ti,ab

AND

'Patient'/exp OR (Patient OR Patients OR Population OR Populations OR Public OR Group OR Groups OR Chronic OR Acute OR Behavior OR Behaviors OR Behavioral OR Behaviour OR Behaviours OR Behavioural):ti,ab

AND

'Outcome Assessment’/exp OR (Outcome OR Outcomes OR Efficacy OR Effectiveness OR Improve OR Improved OR Improvement OR Improvements OR Reduce OR Reduced OR Reduction OR Reductions):ti,ab

AND

meta-analys* OR search:ti,ab OR review:it

**Web of Science**

Refined by: DOCUMENT TYPES: ( REVIEW )

Smartphone OR Smart-phone OR “Smart phone” OR Smartphones OR Smart-phones OR “Smart phones” OR Mobile OR Tablet OR Tablets OR iPhone OR Android

AND

Application OR Applications OR App OR Apps OR Intervention OR Interventions

AND

Utilize OR Utilizing OR Administer OR Administering OR Assist OR Manage OR Management OR Role OR Roles OR Tested OR Increase OR Increased OR Increasing

AND

Patient OR Patients OR Population OR Populations OR Public OR Group OR Groups OR Chronic OR Acute OR Behavior OR Behaviors OR Behavioral OR Behaviour OR Behaviours OR Behavioural

AND

Outcome OR Outcomes OR Efficacy OR Effectiveness OR Improve OR Improved OR Improvement OR Improvements OR Reduce OR Reduced OR Reduction OR Reductions

AND

TOPIC: (meta-analys* OR search OR Searched OR Searches) OR TITLE: (review)
